# The complete mitochondrial genome sequence of the giant clam *Tridacna derasa* (Tridacnidae: Tridacna)

**DOI:** 10.1080/23802359.2018.1501290

**Published:** 2018-08-13

**Authors:** Haitao Ma, Lin Lin, Yuehuan Zhang, Shixi Chen, Wei Shi, Ziniu Yu

**Affiliations:** a Key Laboratory of Tropical Marine Bio-resources and Ecology, Guangdong Provincial Key Laboratory of Applied Marine Biology, South China Sea Institute of Oceanology, Chinese Academy of Sciences, Guangzhou, China;; b South China Sea Fisheries Research Institute, Chinese Academy of Fishery Sciences, Guangzhou, China

**Keywords:** *Tridacna derasa*, mitochondrial genome, phylogenetic relationship

## Abstract

The complete mitochondrial genome of the giant clam *Tridacna derasa* was completely sequenced by high-throughput sequencing method. The total length of the complete mitogenome was 20,760bp, including 13 protein-coding genes, 23 transfer RNA genes, 2 ribosomal RNA genes and a non-coding control region. The base composition of the genome is 28.41% A, 36.92% G, 21.94% G and 12.74% C with a total GC content of 34.68%. Phylogenetic tree based on complete mitogenome sequences revealed that *T.derasa* was closely related to *T.squamosa*, both belonging to the *Tridacna* genus.

Giant clams of the family Tridacnidae are economically and ecologically important coral reef species. The genus *Tridacna* includes nine recognized species, including a newly described cryptic species (Penny and Willan 2014). *Tridacna derasa* is the second largest of the family Tridacnidae and is distributed in the Indo-Pacific region between the Nicobars and Fiji (Yonge 1975). In this study, the complete mitochondrial genome sequences of *T. derasa* were determined using a high-throughput sequencing method for the first time, looking forward to better understanding its systematic evolution and further phylogenetic relationship of Tridacna and Tridacnidae.

The specimen was collected from Huangsha Fishery Market, Guangzhou city, Guangdong province, China (N113.24, E23.11). It was stored in Marine Biodiversity Collection of South China Sea, Chinese Academy of Sciences, Guangzhou, China. The total genomic DNA was extracted following the modified CTAB DNA extraction protocol (Attitalla 2011), followed by library preps and pair-end sequencing (2 × 150 bp) by MiSeq (Illumina, San Diego, CA). Approximately 5,965 Mb of raw data and 5,392 Mb of clean data were obtained, and *de novo* assembled by the SOAPdenovo software (Zhao et al. 2011) with an average of about 280 × coverage.

The mitogenome of *T. derasa* is 20,760 bp in length (GenBank accession number MG755811), shorter than those of *T. squamosa* (20,930 bp) (Gan et al. 2016) and *H. hippopus* (22,463 bp) (Ma et al. 2018), which have the smallest mitogenomes of Tridacnidae reported to date. It contains 13 protein-coding genes (PCGs), 23 transfer RNA genes (tRNAs), 2 ribosomal RNA (*12S rRNA* and *16S rRNA*) genes and a non-coding control region (D-loop). Mitogenome base composition was biased toward A + T content at 65.32% (28.41% A, 36.92% T, 12.74% C, 21.94% G), similar to the mitochondrial genomes of *T. squamosa* and *H. hippopus*. The 13 identified PCGs vary in length from 114 to 1677 bp. Five PCGs initiated with ATT as the start codon, while *ATP6*, *ND2*, *ATP8* and *ND1* began with ATG, *ND4L*, *ND3* and *COIII* started with ATA and *COI* with ATC. Eleven of the PCGs contained a complete (TAA) or incomplete (T––) stop codon and the remaining two genes ended with TAG. The length of the 23 tRNA genes ranged from 62 to 71 bp, and all of the tRNA genes had typical secondary structure. The *12S rRNA* gene was located between *tRNA-Leu* and *ND6*, and was 929 bp long, while the *16S rRNA* gene was located between *tRNA-Ile* and *ND1*, with a length of 1203 bp. A 2831 bp control region (D-loop) was located between *tRNA-Arg* and *COII*, with an A + T content of 66.55%.

A neighbor-joining phylogenetic tree of *T. derasa* and other three closely related species were constructed on the complete mitochondrial genomes using MEGA6 (Tamura et al. 2013) (Figure 1). The result suggested that as expected, *T. derasa* was placed closely to *T. squamosa*, both of which belong to the genus *Tridacna*.

**Figure 1.**
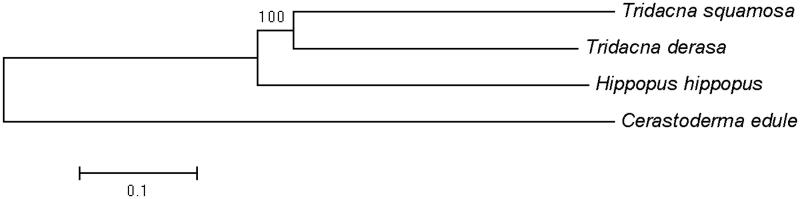
Neighbour-joining phylogenetic tree of *Tridacna derasa* and other three closely related species based on the complete mitochondrial genomes. GenBank accession numbers: *Cerastoderma edule* (MF374632); *Hippopus hippopus* (MG722975); *Tridacna squamosa* (KP205428).
